# Intraindividual Variability in Inhibition and Prospective Memory in Healthy Older Adults: Insights from Response Regularity and Rapidity

**DOI:** 10.3390/jintelligence6010013

**Published:** 2018-03-01

**Authors:** Emilie Joly-Burra, Martial Van der Linden, Paolo Ghisletta

**Affiliations:** 1 Faculty of Psychology and Educational Sciences, University of Geneva, CH-1211 Geneva, Switzerland; Martial.VanDerLinden@unige.ch (M.V.d.L.); Paolo.Ghisletta@unige.ch (P.G.); 2 Swiss Centre for Affective Sciences, University of Geneva, CH-1211 Geneva, Switzerland; 3 Department of Psychology, University of Liège, B-4000 Liège, Belgium; 4 Faculty of Psychology (French), Swiss Distance Learning University, CH-3900 Brig, Switzerland; 5 Swiss National Centre of Competence in Research LIVES—Overcoming Vulnerability: Life Course Perspectives, Universities of Lausanne and of Geneva, CH-1211 Geneva, Switzerland

**Keywords:** intraindividual variability, prospective memory, prepotent response inhibition, Go/NoGo SART task, amplitude of fluctuations, autoregressive parameter, random process fluctuation, functional adaptability, functional diversity

## Abstract

Successful prospective memory (PM) performance relies on executive functions, including inhibition. However, PM and inhibition are usually assessed in separate tasks, and analytically the focus is either on group differences or at most on interindividual differences. Conjoint measures of PM and inhibition performance that take into account intraindividual variability (IIV) are thus missing. In the present study, we assessed healthy older adults’ level of performance and IIV in both inhibition and PM using a classical Go/NoGo task. We also created a prospective Go/NoGo version that embeds a PM component into the task. Using dynamic structural equation modeling, we assessed the joint effects of mean level (μ), an indicator of amplitude of fluctuations in IIV (or net IIV; intraindividual standard deviation, *iSD*), and an indicator of time dependency in IIV (the autoregressive parameter ϕ) in reaction times (RTs) on inhibition and PM performance. Results indicate that higher inhibition failure, but not IIV, predicted PM errors, corroborating the current literature on the involvement of prepotent response inhibition in PM processes. In turn, fastest RT latency (μ) and increased net IIV (*iSD*) were consistently associated with prepotent response inhibition failure, while coherence in RT pattern (ϕ) was beneficial to inhibition performance when the task was novel. Time-dependent IIV (ϕ) appears to reflect an adaptive exploration of strategies to attain optimal performance, whereas increased net IIV (*iSD)* may indicate inefficient sustained cognitive processes when performance is high. We discuss trade-off processes between competing tasks.

## 1. Introduction

### 1.1. IIV as a Tool to Study Cognitive Aging

Aging is associated with a broad range of functional changes in cognition and structural changes in the brain [1]. For decades, the bulk of cognitive and developmental studies have focused on interindividual differences in mean level of performance, regarding reaction time (RT) latency and error rates [2]. In that context, short-term variations in response within the same individual have mainly been considered error measurement, or noise, in the data. Early works from psychologists such as Fiske and Rice, nevertheless, stressed the importance of considering short-term fluctuations as valuable information to further the understanding of interindividual differences [3]. However, the substantial growth of intraindividual variability (IIV) as a tool to describe cognitive functioning, or, as Hultsch and MacDonald put it, “as a theoretical window onto cognitive aging,” is fairly recent [4,5,6]. Because assessing short-term fluctuations requires many repeated assessments for each participant, this recent expansion of IIV as a tool to study human behavior and cognition was largely made possible by advanced statistical tools implemented in software that can handle large datasets.

Two broad types of IIV are often distinguished [7]: dispersion, defined as amplitude of fluctuations across multiple tasks (e.g., variability in global performance across a battery of attentional tasks), and inconsistency, amplitude of fluctuations across trials from the same task (e.g., variability in response latency across the items within a single sustained attentional task). Of these, the latter has probably received more attention in empirical research [4]. The present study focuses on the latter. This within-task trial-to-trial variability, which we will call IIV throughout the paper, has been shown to increase with age [8,9], even after controlling for mean level of performance [10]. Thus, even if individuals of different ages (e.g., younger and older adults) are rendered statistically equivalent with respect to their level of performance, such that there is no longer a difference between their mean performance because of a statistical control, the two age groups remain different with respect to their fluctuations around their means. These fluctuations are at the heart of IIV and thus provide complementary information about cognitive functioning with respect to the mean. Furthermore, IIV is greater in individuals with neurological disorders than in healthy subjects [11] and, far from being noise in the data, has been shown to be a good indicator of the integrity of neuroanatomical functioning in older adults [12,13]. In addition, individual differences in IIV predict level of cognitive performance such that increased IIV in RT has been linked to decreased executive control and resistance to distractor inhibition processes both in the general population and in patients with frontal lesions [11,14]. It has therefore been suggested that increased IIV reflects fluctuant executive control processes along the task and/or sustained attention lapses [14,15]. Moreover, IIV has been shown to predict not only short-term performance within a task, but also long-term cognitive decline [16,17], everyday problem-solving [18], psychopathology [19], and mortality [20,21]. In sum, the detrimental effects of increased IIV are observed on both specific cognitive task performance and more broad functional outcomes. Thus, IIV appears to be a fundamental marker of cognitive aging [22].

### 1.2. Prospective Memory and Cognitive Control in Older Adults

#### 1.2.1. Automatic and Controlled Processes in Prospective Memory

While the relationship between IIV and general age-related cognitive decline has been studied for decades, other research fields in psychology are now turning to the study of IIV to deepen the understanding of cognitive processing underpinning performance. This is currently the case for prospective memory. Prospective memory (PM) is defined as the realization of delayed intentions in response to a given situation or cue [23], and is known to overall decrease with age [24,25,26]. PM enables us to remember to ask a friend a question the next time we see him or to post a letter while passing by the mailbox on our way to work. PM requires four phases: (1) intention formation; (2) intention retention; (3) intention initiation, and (4) intention execution [27]. Critically, PM differs from dual-task or mere action monitoring by (a) the presence of a delay between intention formation and action plan execution, during which the intention leaves the focus of attention; (b) the absence of explicit prompts to switch from the current activity (ongoing task) to the realization of the delayed intention; and (c) the fact that the prospective task is to be performed intermittently and less frequently than the ongoing task [28,29].

The successful realization of delayed intention relies on both prospective and retrospective components of PM. The prospective component is responsible for remembering that something has to be done when a given cue is encountered, while the retrospective component refers to remembering what has to be done. At first, it was suggested that the prospective component, which requires effective cue detection, is supported by automatic processes, while the retrospective component relies on more controlled processes [30]. Automatic prospective retrieval is proposed to rely on a reflexive association between the PM cue and the intended action [31], such that when the cue appears, the intended action is automatically retrieved from memory through recollection. Opposite to that idea is the preparatory attentional and memory process model, which posits that prospective remembering relies on strategic monitoring [32]. This monitoring process involves sustained attention and working memory capacity allocated to detection of the cue, which is no longer available for the ongoing task [33,34]. In line with this idea, brain regions involved in sustained attention and maintained intention are activated in participants expecting a PM cue, indicating anticipatory processing of PM cues, which may reflect monitoring for prospective cue and intention retrieval while performing the ongoing task [35]. From another standpoint, the multiprocess framework states that prospective intention retrieval occurs on either an automatic or controlled basis, depending on a series of task characteristics (importance of the prospective task, nature of the PM cue, its relationship with the intention, degree of absorption in the ongoing task, etc.) and individual differences in personality characteristics. One major determinant of the involvement of controlled monitoring processes is the focality of the PM cue: a task can be either focal, when the target cue feature for PM retrieval is to be processed in the ongoing task, or nonfocal, when the critical feature cuing for the PM task is not directly processed for ongoing task completion. A typical example of a focal PM task is a lexical decision task (word vs. nonword), in which the prospective cue is a certain category of words (e.g., fruits). In contrast, a nonfocal cue can be, for instance, a certain allocated target position on the screen or a particular color of a frame around the item to be processed for the ongoing task [36]. Importantly, the multiprocess framework predicts that nonfocal PM tasks require more controlled processes of strategic monitoring of the PM cues, whereas cue detection in focal PM tasks can efficiently rely on automatic recollection processes.

#### 1.2.2. Age-Related PM Decline and Cognitive Control

Although retrospective memory plays an important role in effective PM performance, it does not appear to explain the observed age differences in performance. Instead, the distinction between controlled and automatic processes seems to be a better candidate to explain age decline in PM. Indeed, age effects are generally more pronounced for controlled than automatic processes [37,38]. Therefore, older adults’ performance is substantially more impaired when attentional load is high and correct task execution requires controlled processes [39,40]. Accordingly, PM impairment with age seems to be particularly exacerbated for nonfocal as compared to focal PM tasks, the former requiring sustained attention and working memory capacity allocation to monitor for the target [31,41,42,43]. However, PM performance that relies on automatic intention retrieval processes can still, to a lesser extent, be affected by age, given that dividing attention between the ongoing task and the prospective requirements may increase the working memory demands anyway [44]. For this reason, Zuber and colleagues recently suggested that controlled and automatic processes always coexist within the same PM task, whether focal or nonfocal [45]. The predominance of control over automatic processes would therefore vary according to the nature of the task and to the PM phase within that task (see also [42]). Even if intention retrieval relied on automatic processes in focal PM tasks, correct intention execution may still require controlled processes.

Considering that retrieving and/or executing the intention entails self-initiating processes, PM processes and several facets of executive functioning can be considered to be somewhat intertwined [45,46,47]. Accordingly, the influence of executive functions on PM performance appears to be more pronounced in older than younger adults [47]. Most of the previous work on the involvement of executive functions in PM performance was based on global measures rather than specific facets of executive functioning [14,47,48]. However, it is now commonly accepted that executive function is not a unitary construct. As demonstrated by Miyake and coworkers, executive functions can be split into related but distinct facets: shifting, updating, and inhibition [49]. Friedman and Miyake showed that inhibition itself can further be divided into three related subprocesses: prepotent (or dominant) response inhibition, distractor-response inhibition, and resistance to proactive interference [50]. More specifically, they showed that prepotent response and distractor response inhibitions are closely related, hence considered a single construct, distinct from proactive interference inhibition. The few studies formally investigating the role of these specific facets of executive functioning in PM processes are therefore relatively recent. More precisely, diminished PM performance seems to be mediated by working memory, prepotent response inhibition, and shifting [43,45,48,51], more so as prepotent inhibition, shifting, and working memory are known to decline with increasing age [40,52,53,54].

#### 1.2.3. The Specific Role of Prepotent Response Inhibition in PM

While the role of working memory (e.g., [51,55]) and shifting [32,56,57] in successful PM has received substantial attention, less is known concerning the specific role of prepotent response inhibition. It has been proposed that prepotent response inhibition plays a role both during monitoring for the cue, because one has to regularly inhibit the ongoing task in order to check for the PM cue [45,56], and during intention execution, because one has to inhibit the current ongoing task to be able to engage in effective execution of the PM intention [58]. Accordingly, Kliegel and colleagues showed that PM performance is particularly impaired in older adults when a task requires high inhibitory control [58].

However, the aforementioned studies focused on mean level of performance on PM and executive control. They therefore provide insights only on the role of interindividual differences in average performance on various facets of executive functions on PM global performance. Another approach to understanding the cognitive processes underlying PM performance is to consider IIV in tasks assessing cognitive processes supposedly involved in PM, such as prepotent response inhibition.

### 1.3. IIV in Prepotent Response Inhibition and PM

Building upon the idea that IIV in cognitive performance may reflect lapses of attention and controlled processes, Manly and colleagues used the Go/NoGo (GNG) task, a sustained attention-to-response task, to relate prepotent response inhibition performance to both mean level and IIV in RT [59]. In this task, sustained attention is required to maintain the task set between trials, while inhibition is needed to stop, or override, the inappropriate dominant response when an infrequent target is shown [60]. Both mean RT and IIV in this task independently predicted inhibition failure. Furthermore, when constraining participants to respond at a given pace (i.e., controlling for mean RT and variations in RT), IIV was still associated with increased error rates, thereby ruling out a speed/accuracy trade-off as the sole reason for inhibition failure. These results further corroborate the claim that IIV reflects fluctuations in executive processes, leading to inhibition failure independent of response latency. Similarly, Bellgrove and co-workers reported that lower IIV in GNG was associated with greater inhibitory performance in young to middle-age adults [61]. Using functional magnetic resonance imaging, they showed that IIV was positively correlated with activation in brain regions involved in sustained attention and inhibition. The authors therefore suggested that IIV reflects a greater demand for executive control in order to maintain task performance. In older adults, similar behavioral results were obtained by Rochat and colleagues [62]. While healthy older adults and older adults with dementia did not differ on their mean RT, IIV in GNG was higher in older adults diagnosed with Alzheimer’s disease than in healthy controls. Critically, inhibition errors in GNG were once again associated with larger variability in RT, even after controlling for working memory capacity and shifting. Together, these studies suggest that variability in RT predicts inhibitory performance beyond the effect of mean-level RT; thus, IIV may reflect cognitive impairments that mean-level RT alone does not reveal.

A few studies very recently investigated IIV as predictive of PM performance. Loft and colleagues reported that a slowing in mean RT on nonprospective trials and increased variability in these RTs are beneficial to prospective performance in a nonfocal, but not in a focal, PM task [36]. Loft and co-workers thus suggested that increased mean RT and variability reflect regular monitoring for the prospective target. In contrast, Haynes et al. consistently found increased IIV in independent working memory and processing speed tasks to be predictive of lower performance on a composite PM score [63]. These authors speculated that the shared association between IIV and PM performance may arise from fluctuations in focus on the task and target monitoring. Similarly, Ihle and colleagues focused on costs to IIV as an outcome variable [64]. Costs to IIV are operationalized as the difference, computed for each participant, in amplitude of fluctuations as indicated by an increase in intraindividual standard deviation (*iSD*) between the pure ongoing task compared to the ongoing task plus embedded prospective task. In other words, costs to IIV assess the extent to which IIV increases when adding the PM task to the ongoing task. Ihle et al. showed that increased costs to IIV in nonfocal PM tasks correlate with lower PM, prepotent response inhibition, and working memory performance; these correlations did not emerge in focal PM tasks. They found no association between costs to mean RT (i.e., increase in mean RT between the pure ongoing task versus the ongoing task with embedded prospective task) and PM performance. Thus, it is not quite clear yet (a) whether IIV in RT reflects adaptive monitoring for target or maladaptive executive-control fluctuations, or (b) the extent to which IIV in prepotent response inhibition specifically predicts PM performance. In that respect, we believe it is useful to consider the distinction between two components of IIV: amplitude of fluctuations and temporal dependency.

### 1.4. Temporal Dependency in IIV

**Figure 1:**
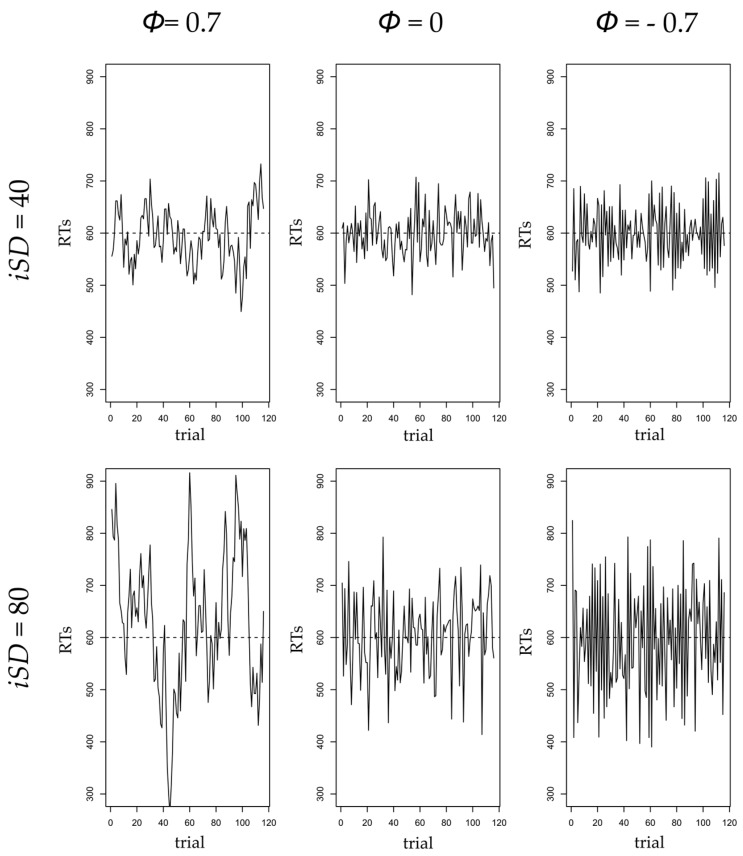
Simulated distributions of time series as a function of the values of the autoregressive parameter (ϕ, representing the time-structured component of intraindividual variation, IIV) and amplitude of fluctuation (intraindividual standard deviation, *iSD*, representing net IIV). From left to right, column panels represent time series for a positive (ϕ = 0.7), null (ϕ = 0), and negative (ϕ = −0.7) autoregressive parameter. Top line panel represents time series for low amplitude of fluctuations (*iSD* = 40), and bottom line panel represents time series for a high amplitude of fluctuations (*iSD* = 80). Mean reaction time (RT) = 600 ms does not vary across time series.

Although amplitude of fluctuations in RT, or the net size of IIV, is unquestionably valuable to study executive control fluctuations and explain cognitive performance, it does not consider the temporal aspects in the data, also called time-structured IIV [65,66]. Whereas amplitude in IIV, or net IIV, captures the range of RTs, time-structured IIV reflects dynamic aspects of cognitive functioning, such as adaptation, regulation, or search for strategies over time. Gilden argued that time dependency in RT is a signature of cognitive complexity and can reflect psychological processes involved in task solving [67]. Autoregressive models are one framework to operationalize temporal dependency in IIV [65,68]. These models assess the effect of the variable of interest at the previous measurement occasion on itself at the present occasion. In the case of RT, a positive autoregressive parameter (ϕ) signifies that if one had a slow RT at a given trial, one is likely to also have a slow RT at the next trial. Conversely, if one had a fast RT at a given trial, one is likely to also have a fast RT at the next trial. Hence, with a positive autoregressive effect, RTs tend to stay above or below one’s mean level of RT for multiple trials before coming back to the mean RT level. The stronger the autoregressive parameter value, the longer it will take for the RTs to return back to the mean level. In other words, when one deviates from mean RT at a given trial, the higher the autoregressive parameter, the longer this deviation is likely to persist [68]. As a consequence, a positive autoregressive coefficient is interpreted as short-term coherence in response pattern, because it indicates alternation between periods of rather slow and rather fast trials in respects to one’s mean RT. For instance, in studies considering day-to-day affective regulation, a positive autoregressive parameter has been interpreted as inertia to return to affective equilibrium [69]. The higher the autoregressive parameter, the longer it took for participants to recover from an emotional shock. In contrast, a null autoregressive parameter indicates that the RT for a given trial is independent from the RT for the previous trial, thereby indicating an absence of response patterns. In the case of affective regulation, this would mean that yesterday’s affect will not impact today’s affect. In other words, a participant with a null autoregressive parameter would display a random pattern of affect scores, hence a total lack of affective regulation. Finally, a negative autoregressive parameter signifies that if one had a slow RT at a given trial, one is likely to have a fast RT at the next trial. Conversely, one would have a slow RT at a given trial when the previous RT was fast. Negative autoregressive parameters, therefore, produce a back-and-forth pattern of RTs with consecutive fast and slow responses, as if participants tried to compensate from one trial to the next. Again, in the case of affective regulation, a negative autoregressive parameter would mean that if one was depressed yesterday, one is likely to feel better today, and probably depressed again tomorrow. A graphic illustration of the impact of autoregressive parameter and amplitude of fluctuations variations on time-series distributions is presented in Figure 1.

Both amplitude of fluctuations and temporal dependency in IIV, therefore, may shed light on complementary information for understanding psychological functioning. Indeed, Ghisletta and colleagues reported that greater amplitude of fluctuations in a simple RT task predicted decline in fluid intelligence two years later, whereas higher consistency in time-structured variability in the same task predicted maintenance of the same outcome two years later [16]. Analyzing longitudinal data on negative affect, Wang and colleagues showed that net IIV predicted health complaints, while time-structured IIV further predicted chronic health problems; interestingly, mean level predicted neither outcome [68]. Similarly, using multilevel autoregressive models, Hamaker and co-workers recently reported that higher time dependency in older adults’ negative affect was positively related to a higher mean level of negative affect, thereby suggesting a detrimental outcome of the carryover effect of negative affect [70]. These and similar results should therefore spur further research aimed at gauging the respective contributions of mean level of performance and both net and time-structured IIV to PM functioning.

### 1.5. Objectives of This Study

The aims of the present work are threefold. First, we seek to extend previous results indicating that IIV in GNG and PM tasks predicts, respectively, inhibition and PM performance in older adults. To do so, we investigated RT in terms of mean level, as well as amplitude of fluctuations in IIV and time-structured IIV modeled within the autoregressive framework. We assessed level of performance and IIV in inhibition performance in 100 healthy older adults using a classical GNG task. We then modified the task to embed a PM component and likewise estimated level of and variability in performance. In line with previous studies, we predicted that faster and more variable RTs would result in increased inhibition errors in GNG. Second, in an exploratory fashion, we compared IIV patterns across the two versions of the tasks. Given that we expected the prospective version of GNG to have a higher demand for controlled executive processes than the classical version of the task, we anticipated slower and more variable RTs in the former than in the latter. Finally, the third aim of this study is to test whether inhibitory performance in the modified GNG task predicts PM performance within the same task, given that previous literature strongly suggests that inhibition mediates age differences in PM [43,47,71]. We expected increased inhibition errors to predict poorer PM performance, even after controlling for working memory capacity. We are not aware of any other study using an inhibition task as an ongoing task for a PM task.

Here, we propose at least two interpretations of the autoregressive parameter (assessing the time-dependent aspect of IIV): on the one hand, coherence in RT patterns (i.e., a strong positive autoregressive parameter) could be associated with a strong activation of task set and better cognitive control, hence better performance in the task. On the other hand, strong coherence in RT patterns could reflect strong activation of the dominant response, rendering successful inhibition of this response less likely. In the first case, we would expect a negative relation between the autoregressive parameter and the number of inhibition and/or PM errors. In the second case, that relation would be positive. In both cases, a positive autoregressive parameter indicates persistence, or consistency, in RT patterns over time.

## 2. Materials and Methods

### 2.1. Participants

The initial sample for this study included 100 French-speaking healthy retired adults over 65 years of age, living independently at home, among the general population (62% women, age: 65–92 years). Exclusion criteria were dementia diagnosis, neurological antecedents, severe motor or sensory disability, and known psychiatric disorders. All participants gave their informed consent to participate. The study was conducted in accordance with the Declaration of Helsinki, and the protocol was approved by the Ethical Committee of the Faculty of Psychology and Educational Sciences at the University of Geneva. Participants were financially remunerated for the study.

Eight participants were excluded prior to statistical analysis, because they either did not complete all the cognitive task analyzed here (2 participants), reported not complying with prospective instructions on purpose (in order to maximize their inhibition performance; 3 participants), pressed the wrong response keys (2 participants), or did not remember the correct prospective cue (1 participant).

### 2.2. Procedure and Material

The present study was part of a wider project using a mixed qualitative and quantitative methodology to assess the nature of the relationships among cognitive functioning, goal-directed behaviors, and well-being in healthy older adults. Participants completed various cognitive tasks, including a classical Go/NoGo task and a modified version of the task in order to include a PM component, the Mini Mental State Examination (MMSE) [72], and Letter-Number Sequencing (LNS, *Wechsler Adult Intelligence Scale III*) [73]. Participants also completed the French versions of the Center for Epidemiologic Studies (CES-D) Depression Scale and the Lack of Initiative and Interest Scale (IIS) as fillers between the two Go/NoGo tasks [74,75]. The purpose was to set a delay between the instructions of the PM task and performance of the task.

### 2.3. Classical and PM Go/NoGo Tasks

The classical Go/NoGo Task (CGNG, also known as Sustained Attention to Response Task, adapted from Rochat et al. [62]), is a computer-based task in which participants have to inhibit a prepotent motor response when an infrequent target stimulus appears on the screen. In this task, participants viewed one digit (between 1 and 9) at a time and were instructed to press the J key on the keyboard when they were shown any digit except 3. When the digit 3 appeared on the screen, participants were instructed to do nothing and wait for the next digit to appear. Each digit was displayed for 1500 ms against a black background, followed by a mask (a white cross in a circle), for 900 ms. Digits varied randomly in font size (5 font sizes between 63 and 135 points) and color (blue, pink, yellow, light green, dark green, gray, brown, and orange); both dimensions were irrelevant in CGNG. Before beginning the task, participants completed an 18-trial practice block that included 4 NoGo (i.e., digit 3) targets. In case of error, they received immediate feedback before the next trial. After the practice block, the CGNG task included 2 blocks of 117 trials each, during which participants did not receive feedback on their performance. Each digit, including 3, was presented 13 times in each block, thus giving a ratio of 104 Go trials (89%) to 13 NoGo trials (11%). Digits appeared randomly, so as to avoid consecutive NoGo trials. Participants were instructed to respond as quickly and accurately as possible. A short break of 5 min separated the two blocks.

After completing the CGNG task, participants were given instructions for the Prospective Go/NoGo (PGNG) task. They were told that now the digit color was relevant for successful task completion. Participants had to continue the previous task (i.e., press the J key when presented with any digit but 3) and additionally perform a second action (i.e., press the space bar after pressing the J key) when a digit in blue appeared (prospective cue). After instruction, participants filled out the CES-D and IIS (not analyzed here), in order to introduce a 10 min delay between prospective instructions and PGNG, preventing rehearsal of the prospective intention in working memory. Participants were not reminded of prospective instructions before beginning the PGNG.

The PGNG also contained 2 blocks of 117 trials each, but no practice block. Each block comprised 91 Go trials (78%), 13 NoGo trials (11%), and 13 Prospective trials (11%) in which prospective items were blue digits. There was no overlap between Prospective and NoGo trials (i.e., no blue digit 3). Again, item order was pseudo-randomized such that there were no consecutive NoGo or Prospective trials.

In order to design the PGNG task based on CGNG, we followed Einstein and McDaniel’s guidelines for the creation of a typical laboratory paradigm for studying PM [76]. These include first presenting participants with instructions for the ongoing task and practicing this task (i.e., CGNG), then giving them the PM instructions (i.e., press the space bar when the digit is blue) and introducing a delay so that the prospective intention leaves the focus of awareness, then, after the delay, reintroducing the task (i.e., PGNG) without reminding the participants of the prospective instructions. Accordingly, CGNG is not a prospective task in itself, because (a) there is no delay between instructions for CGNG and completion of the task, and (b) NoGo trials in CGNG require inhibiting a prepotent response and not remembering to perform an additional action or to replace the regular action from the ongoing task with an alternative prospective action. PGNG, therefore, assesses inhibition performance in the NoGo trials and PM performance in the Prospective trials, where participants have to remember to perform this additional action.

Measures of interest for CGNG were reaction times (RTs) for correct Go trials and number of commission errors (incorrectly pressing the J key on a NoGo trial), the latter reflecting inhibition failure. For PGNG, we considered RTs for correct Go and Prospective trials, number of commission (inhibition) errors, and number of prospective omissions (forgetting to press the space bar on a Prospective trial). RTs were treated as trial-by-trial time series at an intraindividual (within-person) level, whereas commission errors and prospective omissions were summed for each block and treated as scores at an interindividual (between-persons) level. The analyses kept track of the actual trial number (i.e., NoGo trials in CGNG were considered missing and were part of the time series).

### 2.4. Analyses

#### 2.4.1. Data Preprocessing

##### Suppression of Extreme RTs and Log Transformation

The first trial for each block was excluded from analysis, given that it consistently presented a very high RT. On the remaining 116 trials, RTs of correct trials below or above 3 standard deviations from the intraindividual mean were excluded from analysis and replaced with missing values for each subject (1.18% and 1.51% of trials in CGNG and PGNG, respectively). RTs were log-transformed to reduce skewness [77,78,79]. We thus obtained a total of 368 time series across 92 participants: four time series of log(RTs) for each subject, corresponding to blocks 1 and 2 of both CGNG and PGNG.

##### Amplitude of Fluctuations (iSD) Calculation and Stationarity Assessment

In order to compute an indicator of intraindividual amplitude of fluctuations, also referred to as net IIV [66], within each block, we calculated the intraindividual standard deviation (*iSD*) of log(RTs) for each individual’s time series. *iSD* reflects the amount of fluctuation for an individual but does not consider the temporal dynamics of the fluctuation.

While *iSD* provides information on the amplitude of intraindividual fluctuations, time-series analyses further model the time dependency in the data, or time-structured IIV [66], via autoregressive models. In other words, a time-series analysis tests whether the RT at time *t* (from 1 to *T*) is influenced by previous RTs at time *t-k*, thereby capturing the temporal dynamic effects in the data up to a lag *k* (*k* < *T*) (for further details, see Section 2.4.2). However, time series can display general trends, such as learning effects across trials, resulting in decreasing RTs (negative trends), or fatigue effects, resulting in increasing RTs (positive trends). Such trends are known to potentially bias time-dependency estimation and thus must be removed from the data [68]. To screen for possible trends in the data, we visually inspected the time series and performed the Augmented Dickey-Fuller (ADF) test for each of the 368 time series [80]. This test formally assesses whether time series are stationary around zero, around a constant value (mean), or around a constant value plus a regular change pattern (mean and trend). The ADF test assesses the constancy of the mean and the variance of the dependent variable across repeated assessments. In our data, we expected the time series to be stationary around a mean (i.e., the mean RT). In the case of stationarity around a mean plus a trend, data must be detrended prior to any further analysis.

#### 2.4.2. Dynamic Structural Equation Modeling with First-Order Autoregressive Parameter

Classically, autoregressive models are used to model time dependency in single-case time series. Such models estimate the lagged effects of RTs at previous trials *t-k* on the current RT at trial *t.* Autoregressive models thus estimate a *k*-order autoregressive parameter $\phi_{k}$ such that $RT_{t}=\phi_{1}RT_{t-1}+\cdots \;+\phi_{k}RT_{t-k}+\;\varepsilon_{t}$. For the sake of simplicity, we will limit analysis in this study to first-order autoregressive vectors (also called at lag 1, when *k* = 1), estimating the direct effect of RT at time *t-*1 on RT at time *t*. Practically, the autoregressive parameters of order 1 estimated here indicate to what extent the RT at a given trial depends on the RT at the previous trial. As previously mentioned, a positive autoregressive parameter $\phi_{k}$ indicates a manifestation of coherence in response patterns across time (if one had a fast RT at trial 1, one would also tend to have, on average, a fast RT at trial 2), while a negative parameter reflects erraticism in responses (if one had a quite fast RT at trial 1, one would tend to have, on average, a rather slow RT at trial 2) (see [30,46]).

Autoregressive models are classically estimated separately for each individual time series. Here, this would imply estimating over 300 autoregressive models. A new approach allowing for the simultaneous estimation of multiple time series is called dynamic structural equation modeling (DSEM) and has recently been implemented in version 8 of Mplus software [81]. DSEM acknowledges the nested structure of the data by providing a multilevel extension of autoregressive models [70], in which trials (within-person, level 1) are nested within individuals (between-person, level 2), and allows for the inclusion of level-2 predictors and outcome variables in the model. In practice, these models can conjointly estimate both group effects and between-person variations in mean RT latency and net and time-structured IIV, while allowing these dimensions to predict other outcome variables such as accuracy or errors in the task. DSEM also has the advantage of taking into account missing values, which is typically not the case in most statistical software proposing time-series analyses. This feature is particularly important in the GNG task, as all correctly answered NoGo trials are followed by a missing RT (i.e., participants inhibit the prepotent motor response of pressing the J key when the number 3 appears). These models are implemented in a Bayesian framework such that parameter estimates are obtained from posterior distributions (based on noninformative priors following a normal distribution *N*(0, 10^10^)) and inferential conclusions are drawn based on the credible intervals (CIs) of these posterior distribution.

We follow Hamaker and colleagues [70,82,83] and Asparouhov and colleagues [84] to present DSEM. Conceptually, the model first decomposes a RT into within- and between-person components as follows:

(1) $$RT_{it}=\mu_{i}+RT_{it}^{*},$$

where $\mu_{i}$ is the time-invariant (between-person) mean RT for individual *i*, while $RT_{it}^{*}$ represents the individual (within-person) deviations from $\mu_{i}$ at trial *t*.

The within-person component $RT_{it}^{*}$ is decomposed as in the following equation:

(2) $$RT_{it}^{*}=\phi_{i}RT_{i,t-1}^{*}+\zeta_{it},$$

where $\phi_{i}$ is the first-order autoregressive parameter of individual *i* for RTs for two successive trials and $\zeta_{it}$ is the residual representing the variations in RT at trial *t* not explained by RT at trial *t-*1. These residuals are supposed to be normally distributed around zero with constant variance $\sigma_{\zeta }^{2}$. Both the overall mean $\mu_{i}$ and the autoregressive parameter $\phi_{i}$ are allowed to vary across persons (hence the subscript *i*). That is, they both have random effects (υ), as in

(3) $$\begin{array}{l} \mu_{i}=\;\gamma_{\mu }+\upsilon_{\mu i},\\ \phi_{i}=\gamma_{\phi }+\upsilon_{\phi i}, \end{array}$$

where $\upsilon_{\mu i}$ and $\upsilon_{\phi i}$ are normally distributed, have constant variance $\sigma_{\mu }^{2}$ and $\sigma_{\phi }^{2}$, respectively, and are allowed to covary ($\sigma_{\mu \;\phi }$) with each other.

As is customary in multilevel modeling, we can combine Equations (1) to (3) to obtain

(4) $$RT_{it}=\gamma_{\mu }+\gamma_{\phi }RT_{i,t-1}^{*}+\upsilon_{\mu i}+\upsilon_{\phi i}RT_{i,t-1}^{*}+\;\zeta_{it},$$

where $\gamma_{\mu }$ and $\gamma_{\phi }$ are the fixed effects of the mean RT (i.e., mean RT averaged across participants) and the first-order autoregressive parameters (i.e., the mean autoregressive parameter on the whole sample), respectively. In turn, parameters $\upsilon_{\mu i}$ and $\upsilon_{\phi i}$ indicate the random effects of mean RT level and first-order autoregressive parameter, respectively (between-person variations in mean RT and autoregressive parameter at lag 1, respectively). To investigate the relationship between dimensions of IIV (amplitude of fluctuations and temporal dependency) and level of performance, we added *iSD*s to the model and allowed it to correlate with $\phi_{i}$ and $\mu_{i}$.

In the next step, we make full use of the strengths of DSEM by expanding the autoregressive model and considering additional between-person covariates. We therefore specify the overall traitlike mean $\mu_{i}$, the autoregressive parameter $\phi_{i}$, and the *iSD_i_* to predict commission errors. Hence, level-2 outcome variables can be regressed on fixed effects as follows:

(5) $$CE_{i}=\beta_{0}+\beta_{\mu }\mu_{i}+\beta_{\phi }\phi_{i}+\beta_{iSD}iSD_{i}+\upsilon_{ei},$$

where $CE_{i}$ is the total number of commission errors during a block for individual *i*, $\beta_{0}$ is the intercept, $\beta_{\mu },\;\beta_{\phi }$, and $\beta_{iSD}$ are the regression weights of $CE_{i}$ on mean level RT ($\mu_{i}$) autoregressive parameter $\phi_{i}$ (estimated in Equation (3)), and amplitude of fluctuations (*iSD_i_*), respectively. Finally, $\upsilon_{ei}$ are the prediction residuals. We applied this model to each block of both task versions.

We computed successive DSEMs in order to test our hypothesis. First, single-block models were estimated to (a) assess the respective contributions of $\mu_{i}$, $iSD_{i}$, and $\phi_{i}$ in RT decomposition in each block of CGNG and PGNG, respectively (models M1 to M4); and (b) regress inhibition errors on the fixed effects of these parameters (models M5 to M8). Second, a two-block model (model M9) was computed to (c) estimate relations between the respective $\mu_{i}$, $iSD_{i}$, and $\phi_{i}$ from the second block of CGNG (CGNG2) and the first block of PGNG (PGNG1) and (d) regress prospective omissions in PGNG1 on $\mu_{i}$, $iSD_{i}$, and $\phi_{i}$ and inhibition errors from CGNG2. Finally, given that PGNG had a higher cognitive load than CGNG, we estimated a last two-block model (model M10) to (e) rule out a possible confounded effect of general working memory resources (LNS score) in the prediction of PM errors by inhibition errors.

**Figure 2:**
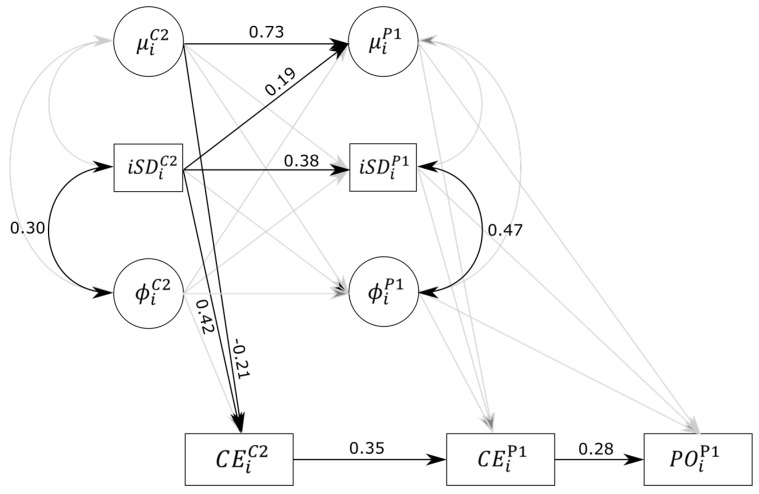
Path diagram for model M9 results. Variables represented in circles are parameters estimated within the dynamic structural equation modeling (DSEM) based on individual RTs, namely mean-level RT ($\mu_{i}$) and the autoregressive parameter of order 1 ($\phi_{i}$) in Classical Go/NoGo (CGNG)2 and Prospective Go/NoGo (PGNG)1. Variables represented in rectangles are the intraindividual amplitude of fluctuations (*iSD_i_*), the total number of commission errors in CGNG2 and PGNG1 ($CE_{i}^{C2}$ and $CE_{i}^{P1}$), and the total number of prospective omissions in PGNG1 ($PO_{i}^{P1}$). Parameters $\mu_{i}$, *iSD*i, and $\phi_{i}$ are regressed on one another across blocks and allowed to covariate within blocks. Finally, error variables are regressed on $\mu_{i}$, *iSDi*, and $\phi_{i}$ in their respective blocks. Single-headed arrows represent regression weights, double-headed arrows indicate covariances. Significant parameters and their corresponding standardized estimates are reported in black. Nonsignificant parameters are depicted in gray. Variances for each variable are not depicted but were nevertheless estimated and were all significant. $R_{\mu^{P1}}^{2}$ = 0.58, $R_{iSD^{P1}}^{2}$ = 0.18, $R_{\phi^{P1}}^{2}$ = 0.16, $R_{CE^{C2}}^{2}$ = 0.25, $R_{CE^{P1}}^{2}$ = 0.24, $R_{PO^{P1}}^{2}$ = 0.13.

We fitted the same model (cf. Equations (1)–(4)) for each block of both versions of the tasks in models M1 to M4 and then included Equation (5) in models M5 to M8. In model M9, Equation (5) was estimated twice, to predict commission errors for CGNG2 and PGNG1. $CE_{i}^{P1}$ (commission errors in PGNG1) was additionally regressed on $CE_{i}^{C2}\;$(commission errors in CGNG2), while $PO_{i}^{P1}$ (prospective omissions in PGNG1) was regressed on $\mu_{i}^{P1}$, $\phi_{i}^{P1}{}_{\;}$, $iSD_{i}^{P1}$, and $CE_{i}^{P1}$. Model M9 is represented in Figure 2. Finally, in model M10, $CE_{i}^{C2}$, $CE_{i}^{P1}$, and $PO_{i}^{P1}$ were additionally regressed on the LNS score.

## 3. Results

### 3.1. Stationarity

According to the ADF test, none of the time series for RT displayed a trend, indicating an absence of global speeding or fatigue effect across trials within each block. On the 368 time series, 363 were stationary across a constant nonzero mean, indicating fluctuations of RT around a within-person mean for each block. Surprisingly, five time series were found to be stationary around zero in PGNG, suggesting that the RTs in these time series vary around a mean not statistically different from zero, which is virtually impossible. Visual inspection of the time series revealed that these series presented extreme variations in RT, with a few abnormally short and long RTs across the block. We therefore excluded the five participants whose time series were stationary around zero from subsequent analysis.

### 3.2. Sample Characteristics and Descriptive Statistics

The final sample for analysis thus comprised 87 older adults (60.92% women). The average age of the participants was 72.44 years (*SD* = 5.01, range = 65–84). Participants had a relatively high number of years of education (*M* = 13.30, *SD* = 2.78), normal general cognitive performance (MMSE mean [*M*] = 27.77, *SD* = 1.86), and globally self-rated their health as good (*M* = 4.00, *SD* = 0.72, on a 5-point Likert scale from 1 = Very bad health to 5 = Very good health). The mean score for LNS was 9.41 (*SD* = 2.36).

### 3.3. Error Patterns

Commission errors and prospective omission rates in the respective blocks of CGNG and PGNG are displayed in Table 1. A Wilcoxon signed-ranks test indicated that summed commission errors were significantly higher in PGNG than in CGNG (*Z* = −7.52, *p* < 0.001). Pooled across blocks, participants failed to inhibit 8.5% of incorrect dominant responses in CGNG, which is comparable to what was reported in Rochat et al. for healthy older adults [62]. Inhibition failure increased to 23.4% in PGNG. Within CGNG, commission errors did not significantly differ between the two blocks (Z = −0.51, *p* = 1.00), while there were significantly more commission errors in PGNG1 than in PGNG2 (Z = −4.41, *p* < 0.001). Concerning PM, overall performance was extremely high, with a 91% mean accuracy pooled across blocks, and prospective omission errors were higher in PGNG1 than in PGNG2 (Z = −3.84, *p* < 0.001; *p*-values were multiplied by the number of tests to correct for type I error bias). Participants therefore had more trouble inhibiting a dominant response in PGNG than in CGNG and, furthermore, showed worse inhibition and prospective performance in the first block than in the second block of PGNG. This suggests some sort of learning effect, or calibration of performance, for the prospective version of the task.

### 3.4. Estimation of DSEMs

Following Hamaker et al. [70,82,83], we estimated DSEMs with a Gibbs random walk sampler algorithm for the Markov chain Monte Carlo estimation running two parallel chains. Each model was estimated using 50,000 iterations and a thinning of 10, resulting in a 5000-iteration–based solution. All models satisfyingly converged according to potential scale reduction statistic values very close to 1.0 (highest value was 1.08 for model 5), indicating convergence of results for the two chains, and symmetric posterior distributions of all parameters, satisfying posterior parameter trace plots and rapidly decreasing to 0 autocorrelation plots for each parameter. Diagnostic plots were not initially satisfying for models 1, 9, and 10. For these, we increased the number of iterations to 250,000 and used a thinning of 50, still resulting in a 5000-iteration–based solution. All models presented satisfying diagnostic plots at the end, indicating stability of the final solutions.

#### 3.4.1. Single-Block Models

##### Fixed and Random Effects

Fixed and random effects estimates for models M1 to M4, along with their 95% CIs, are reported in Table 2. As expected, fixed effects for both mean RT ($\gamma_{µ}$) and amplitude of fluctuations $\left(\gamma_{iSD}\right)$ were consistently higher in PGNG ($\gamma_{µ}^{P1}$ = 6.38 and $\gamma_{µ}^{P2}$ = 6.36, corresponding to 589.93 ms and 561.16 ms, respectively, $\gamma_{iSD}^{P1}$ = 0.20 and $\gamma_{iSD}^{P2}$ = 0.18) than in CGNG ($\gamma_{µ}^{C1}$ = 6.25 and $\gamma_{µ}^{C2}$ = 6.22, corresponding to 518.01 ms and 502.70 ms, respectively, $\gamma_{iSD}^{C1}$ = 0.15 and $\gamma_{iSD}^{C2}$ = 0.14), as suggested by nonoverlapping 95% CIs. This strongly suggests that participants were overall slower and more variable in their RTs in PGNG than in CGNG. These results are consistent with previous findings from Ihle and colleagues indicating that adding a prospective task to the ongoing task comes with a cost, in terms of both mean RT and amplitude of fluctuations [64]. Estimates for the autoregressive parameter $\gamma_{\phi }$ were positive and significant in all models. They appeared higher in CGNG ($\gamma_{\phi }^{C1}$ = 0.30 and $\gamma_{\phi }^{C2}$ = 0.27 for blocks 1 and 2, respectively) than in PGNG ($\gamma_{\phi }^{P1}$ = 0.23 and $\gamma_{\phi }^{P2}$ = 0.14, respectively). Estimated means for ϕ suggest that RT patterns were less consistent in time in PGNG than in CGNG, and that there was even less consistency in the second block of the prospective version of the task.

All random effects were significant, indicating that there were substantial between-person differences in mean RT, amplitude of fluctuations, and time dependency. Such nonzero random effects prompted the investigation of covariances among these parameters.

##### Correlation Estimates

Random effects were allowed to covary at level 2. We report standardized estimates of these covariations and interpret these effects as correlations between the random effects. Correlations between the random effects of mean RT, $\upsilon_{µi}$, and the autoregressive parameter,$\;\upsilon_{\phi i}$, as well as between mean RT and *iSD*, $\upsilon_{iSDi}$, did not significantly differ from 0. The latter reveals that net variability is independent from response latency in this study, which is in line with Bellgrove and co-workers’ results [61]. This consistent independence of mean level of RT and amplitude of fluctuation in our data supports the claim that a linear relation between mean RT and *iSD* should not be arbitrarily assumed [85]. Instead, the two parameters need to be considered for their respective contribution to performance [59,86]. However, a positive correlation between the random effects of the autoregressive parameter and *iSD* was significant in both blocks of CGNG ($r{\left(\upsilon_{\phi i}-\upsilon_{iSDi}\right)}^{C1}$ = 0.65 and $r{\left(\upsilon_{\phi i}-\upsilon_{iSDi}\right)}^{C2}$ = 0.29) and the first block of PGNG ($r{\left(\upsilon_{\phi i}-\upsilon_{iSDi}\right)}^{P1}$ = 0.33), but not in the second block of PGNG ($r{\left(\upsilon_{\phi i}-\upsilon_{iSDi}\right)}^{P2}$ = −0.11). These positive correlations suggest that the participants with more variability were also more consistent in their pattern of RTs from one trial to the next, except in PGNG2. Hence, consistent response patterns across time seem to emerge in participants whose RTs vary more widely around a mean level. A somewhat rushed conclusion would be that amplitude of fluctuations and time-dependency components of IIV are collinear in these data.

##### Prediction of Commission Errors

Models M5 to M8 further included the prediction of commission (inhibition) errors $CE_{i}$ on the previously mentioned fixed effects. This extension of the previous models allows testing whether indicators of level (*μ*) and IIV (*iSD* and ϕ) during performance of the task are related to the total number of commission errors. Estimates for the fixed and random effects of *μ*, *iSD*, and ϕ, as well as the covariances between these random effects, were virtually identical to those estimated in models M1 to M4. Thus, we only report estimates for the regression weights in Table 3 (the full version of the table, with all estimates, is available in Appendix A, Table A1). In all models, $\mu_{i}$ significantly and negatively predicted commission errors, which is consistent with the concept of speed/accuracy trade-off: the higher the RT, the fewer the errors, and the lower the RT, the more numerous the errors. Concerning net IIV, $iSD_{i}$ was positively related to $CE_{i}$ in CGNG1, CGNG2, and PGNG1. The effect just failed to reach significance in PGNG2 (95% credible interval (−0.00 to 0.40)). Hence, the faster and more variable the participant, the more likely inhibition errors were committed in both versions of the task, independent of the general response speed. These results replicate previous findings [59,61,62], and therefore corroborate the claim that increased amplitude of fluctuations in RT negatively impacts performance in a GNG task, reflecting fluctuations in controlled executive processes.

In turn, the autoregressive parameter ϕ negatively predicted $CE_{i}$ in CGNG1 only, suggesting that in the first block of CGNG, participants with higher consistency in their RTs committed fewer errors. In this block, the three parameters together explained 45% of the variance of commission errors. Mean level RT, net IIV (*iSD*), and time-dependent IIV (ϕ), therefore substantially predicted almost half of the inhibition failure variance. This effect size estimate dropped to 25%, 16%, and 17% in models 6, 7, and 8, respectively.

#### 3.4.2. Two-Block Models

##### Coherence of Fixed Effects across Both Versions of the Task

In model M9, all three PGNG1 parameters related to mean RT, amplitude of fluctuations, and time dependency were regressed on the analogous parameters of the CGNG2 block. This model allows testing whether performance (in terms of level and IIV components) during the classical version of the task predicts performance in the prospective version. Parameters within each block were allowed to correlate with each other and to predict commission errors in their respective block. Furthermore, the PGNG1 parameters were allowed to predict the prospective omission errors in the same block. Finally, commission errors in PGNG1 ($CE^{P1}$) were regressed on the same errors in CGNG2 ($CE^{C2}$), and prospective omissions ($PO^{P1}$) were regressed on commission errors in PGNG1. Significant standardized estimates for model M9 are reported in Figure 2. Results indicate that $\mu^{P1}$ was significantly predicted by $\mu^{C2}$ and $iSD^{C2}$ (the respective regression weights are 0.73 (0.59 to 0.84) and 0.19 (0.02 to 0.34)), but not by $\phi^{C2}$ (−0.05 (−0.26 to 0.16)). This indicates a certain degree of traitlike stability of mean RT across the two versions of the tasks, and the more variability in CGNG2, the slower in PGNG1. $iSD^{P1}$ was only significantly predicted by $iSD^{C2}$ (0.38 (0.17 to 0.58)), indicating a moderate relationship between amplitude of fluctuations in CGNG2 and PGNG1. Finally, neither $\phi^{C2}$, $\mu^{C2}$, or $iSD^{C2}$ significantly predicted $\phi^{P1}$, indicating unrelated time-dependency parameters between the two versions of the task. In other words, the fact that one tends to deviate from one’s mean level of RT for a long period of time in the last part of the CGNG does not necessarily imply that the same deviations will be observed when a prospective requirement is added to the task.

As seen previously in model M6, commission errors in CGNG2 ($CE^{C2}$) were still significantly predicted by $\mu^{C2}$ and $iSD^{C2}$ (−0.21 (−0.41 to −0.01) and 0.42 (0.21 to 0.60), respectively; *R*^2^ = 25%). In turn, $CE^{C2}$ did positively predict $CE^{P1}$ (0.35 (0.14 to 0.53), *R*^2^ = 24%), while, unlike in model 7, $\mu^{P1}$ and $iSD^{P1}$ effects just failed to reach significance after controlling for the effect of commission errors in the previous block (−0.18 (−0.40 to 0.04) and 0.18 (−0.07 to 0.42), respectively). In both blocks, even though there seemed to be a stable inhibition ability trait, three-quarters of the variance of inhibition errors in PGNG were not explained by the previous inhibition performance at a simpler version of the task. We interpret this unexplained variance at least partially in terms of ongoing task cost on the inhibition task.

Similarly to models M2, M3, M6, and M7, only the correlations between respective random effects of the *iSD* and the autoregressive parameter ϕ were significant (0.30 (0.03 to 0.53) and 0.47 (0.16 to 0.72) for CGNG2 and PGNG1, respectively).

##### Prospective Errors Prediction

As expected, $CE^{P1}$ significantly predicted $PO^{P1}$ (0.28 (0.06 to 0.49)), suggesting that the more inhibition failures, the more prospective failures as well. None of the effects of mean RT, amplitude of fluctuations, or autoregressive parameter further significantly predicted prospective omissions. As a whole, the model predicted 12.7% of the variance of $PO^{P1}$.

##### Controlling for Working Memory Performance

Finally, in model M10, we added three predictors to model M9. We included predictions from the total score at the LNS task onto $CE^{C2}$, $CE^{P1}$, and $PO^{P1}$. LNS score significantly predicted $CE^{P1}$ (−0.20 (−0.38 to −0.00), *R*^2^ = 29%), but failed to predict $CE^{C2}$ and $PO^{P1}$ (−0.14 (−0.32 to 0.06), *R*^2^ = 28%, and −0.05 (−0.26 to 0.17), *R*^2^ = 14%). This result confirms our expectation that PGNG is more taxing on cognitive resources than CGNG, and further suggests that inhibition performance suffers from limited working memory capacity while prospective performance does not. This is also in line with the claim that monitoring for prospective cues is taxing on working memory capacity, which is no longer available for successful completion of the ongoing task. Of importance, the regression weight from $CE^{P1}$ to $PO^{P1}$ remained significant (0.27 (0.03 to 0.49), *R*^2^ = 14%), indicating that the relationship between inhibition and prospective failure is not due to a general confounded effect of working memory resources.

## 4. Discussion

In the present work, we considered healthy older adults’ performance in both a classical and prospective version of a GNG task. Using DSEM [70,82,83], we gauged the respective contributions of mean level RT, net IIV, and time-structured IIV to inhibition and prospective performances. To our knowledge, this study is the first to use an inhibition task as an ongoing task for a PM task and to investigate time-structured IIV in inhibition and PM.

### 4.1. Inhibition, PM Performance, and Ongoing Task Costs

As hypothesized, inhibition performance in PGNG positively predicted PM performance in the same block. The better the inhibition performance, the higher the prospective accuracy. Importantly, recent work has suggested that age-related deficits in the GNG task could be attributed to impairments in the ability to maintain multiple task sets rather than actual prepotent inhibition impairments [53]. In our data, the link between prepotent inhibition and PM performance does not appear to be due to a confounded general lack of cognitive resources, because the effect was still significant when controlling for working memory performance (see also [54]). Therefore, this effect concurs with previous results from the PM literature indicating that prepotent response inhibition contributes to successful cue retrieval and intention initiation in PM [43,45,47,58]. The present relationship between inhibition and PM is not merely due to ongoing task costs in our data. Had it been the case, we would have observed a negative, instead of a positive, relationship between inhibition and PM errors.

Still, in line with the notion of competing resource allocation between ongoing and PM tasks, inhibition (commission) error rates increased in PGNG compared to CGNG, which indicates ongoing task costs in terms of accuracy on the inhibition (ongoing) task [87,88]. While ongoing task performance decreased in PGNG compared to CGNG, prospective performance was particularly high in the present study [88]. Therefore, error patterns suggest that, at least at the group level, participants prioritized the prospective task at the expense of the ongoing inhibition task. The ongoing task cost is further substantiated by increased mean-level RT and amplitude of fluctuations in PGNG compared to CGNG. Inasmuch as increased net IIV is often considered to reflect greater demand for executive control in exchange for maintained task performance [61,89], results confirm that PGNG is more taxing on executive resources than the classical version of the task [64,90].

### 4.2. Absence of Direct Link between PM Performance and IIV

In the present work, we extended the examination of IIV on inhibition and PM performance by taking into account both the amplitude of net IIV and its time structure, operationalized as the autoregressive parameter ϕ. Regarding PM performance, contrary to our expectations and to previous studies [36,64], neither mean-level RT or net or time-dependent IIV significantly predicted prospective omissions. We cannot tie this absence of effects to the fact that we controlled for the effect of commission errors. As a matter of fact, despite removing commission errors from the model, effects of mean-level RT and net and time-dependent IIV on prospective omission still failed to reach significance. We therefore discuss three nonmutually exclusive possible accounts for the absence of relations between IIV and PM performance.

First, as mentioned previously, PM performance was particularly good in this study (91% accuracy). Loft and colleagues, who did observe a relationship between IIV and PM performance, had accuracy ranges sensibly lower than that observed in our data [36]. We therefore may have failed to observe a link between PM performance and IIV in our data due to a restricted range in the number of prospective omissions. However, the fact that inhibition successfully predicted PM errors tends to invalidate this hypothesis. Indeed, there may be a narrow range of PM errors, but inhibition performance was able to successfully predict at least one part of its variance.

Second, and related to the first point, such high PM performance tends to suggest that our PM task is more demanding than the CGNG, but still not extremely demanding. Thus, the PM task would principally rely on automatic cognitive processes in both cue detection and intention execution. In effect, the multiprocess framework [31,91] states that demands in controlled monitoring processes vary according to certain characteristics of the PM task, among them target cue focality. In the present study, PGNG was designed as a nonfocal task, because the prospective cue, digit color, was irrelevant for the ongoing task. Such nonfocal tasks are expected to predominantly rely on controlled processes. However, the accuracy rate in our data is rather in line with those of studies in which prospective retrieval appeared to rely on spontaneous retrieval [90,92] rather than on controlled processes. Critically, albeit being nonfocal, our PM cue, color, was directly presented at the center of attention (i.e., on the number to be processed for ongoing task) and is a very basic feature to be processed [93]. Importantly, according to the multiprocess framework, such highly distinctive cues can trigger automatic recollection of the prospective intention without the need for strategical monitoring [91]. Hence, nonfocal tasks using highly distinctive PM cues can ultimately present the same pattern of results as focal tasks. Accordingly, the PM accuracy rate in our data is also more in line with the accuracy reported by Loft et al. for their focal task (85%) rather than their nonfocal task (60%). Of importance, effects of net IIV on PM performance were not observed for focal tasks in either Loft and colleagues or Ihle and colleagues [36,64]. Therefore, the absence of effects of both net and time-dependent IIV on PM performance in the present data eventually is in line with previous findings from these authors.

The third account for the absence of IIV effect on PM in the first block of PGNG performance revolves around interindividual differences in task-set prioritization and search for strategies. Participants may have differed in terms of which task they prioritized over the other. In that respect, IIV may, in the first block of PGNG, reflect different cognitive processes in different people, therefore blurring the relation between IIV and PM performance.

### 4.3. Adaptive and Nonadaptive Aspects of IIV and Inhibition Performance

In contrast to the absence of effects on PM, mean-level RT and IIV did predict inhibition performance. Faster RTs and higher *iSD*s consistently predicted increased inhibition errors, whereas higher autoregressive parameters ϕ predicted fewer errors in the first block of CGNG only. In this block, a higher autoregressive parameter was associated with fewer commission errors. Hence, the more coherent the RT pattern, the better the inhibition performance. Interestingly, although their respective influences on inhibition performance in the first block of CGNG went in opposite directions, net and time-dependent IIV correlated positively and strongly. That is, consistent response patterns across time seem to emerge in participants whose RTs vary more widely around a mean level. The more variable participants are, the more consistent their pattern of RT from one trial to the next, or the longer they tend to deviate from their mean RT. To disentangle respective effects of net IIV and time-dependent IIV, we additionally computed a model in which we excluded *iSD* as a predictor of inhibition failure (estimates for this additional model are reported in Appendix B, Table A2). In this model, ϕ did not significantly predict the number of errors. This suggests that higher consistency (as assessed with the autoregressive parameter ϕ) is beneficial to inhibition performance only when portioning out the confounded negative effects of net IIV.

While initially counterintuitive, these effects are compatible with the distinction made by Li and colleagues between adaptive and nonadaptive IIV [94]. In our opinion, this distinction is a key element to apprehend the meaning of IIV indices with regard to the global performance pattern in our data. Li and co-workers propose that detrimental random process fluctuations, indicating a lack of robustness in cognitive processes, are observed when participants arrive at an asymptotic level of functioning (i.e., when they are close to optimal performance). In contrast, adaptive types of IIV include functional diversity and adaptability. The former refers to variability reflecting adaptive exploratory behavior and search for strategies during acquisition of a novel task (i.e., before reaching an asymptotic level of functioning). An example of functional diversity was provided by Siegler [95], who showed, in his pioneering work, that increased IIV in children was associated with exploration of various strategies for successful problem-solving. Functional adaptability refers to the ability to alter functioning in response to perturbations, such as sudden fluctuations in processes or more cognitively demanding tasks, in order to restore optimal functioning. In line with this decomposition of IIV, Allaire and Marsiske reported that net IIV proved to be detrimental in tasks in which older adults already performed close to optimal performance, whereas net IIV appeared to be adaptive in more difficult tasks where exploration of various strategies enabled performance improvement along the task [96].

In our data, a higher autoregressive parameter (higher consistency in RT patterns) is beneficial to inhibition performance and can therefore be interpreted in terms of functional diversity. The fact that this effect only appeared in the first block of the task suggests that the autoregressive parameter here reflects exploration of speed/accuracy trade-off strategies in order to reach optimal performance (cf. [97] for a review of speed/accuracy trade-off). After reaching optimal equilibrium, the overall performance in CGNG, where participants have only one task to complete, is very high, and further exploration of strategies may not be beneficial anymore. In this context, the negative effect of *iSD* on inhibition performance can therefore be related to what Li et al. labeled as the detrimental effect of random process fluctuation [94]. This refers to the assumption that higher net IIV on a trial-to-trial basis reflects fluctuation in executive controlled processes underpinning successful completion of the task [14].

If a higher autoregressive parameter (higher consistency in RT patterns) reflects exploration of strategies in order to master the task, as indicated by results in the first block of CGNG, we would have expected a similar pattern in the first block of PGNG, when the prospective task was added to the GNG. Results indicate that the autoregressive parameter’s effect on inhibition performance just failed to reach significance in PGNG1. A possible explanation is that after two blocks of CGNG, participants were quite familiar with the task, and adjustment to the additional task in PGNG would probably not require an entire block of trials. To test this hypothesis, we split PGNG1 into two time series (trials 1–58 and 59–107) and applied the same model (model 7) to each half-block (results are presented in Appendix B, Table A3). As expected, the autoregressive parameter ϕ was sensibly higher in the first half of the first block of PGNG than in the second half, and also significantly predicted fewer inhibition errors, which was no longer the case in the second half of PGNG1. Similar to what was observed for CGNG1, a higher autoregressive parameter predicted less inhibition failure, whereas increased amplitude of fluctuations predicted more inhibition errors. These results reinforce our proposition that a high autoregressive parameter at the beginning of a task reflects strategy exploration and learning of the task, which we can also interpret as functional adaptability.

Indeed, in PGNG, executive demands of the task increase and participants have to balance the two task sets. Increased variability may become useful when trying to juggle both tasks’ requirements. In the first block of PGNG, participants may try to continue using the same, now counterproductive, response strategy they used in CGNG, which results in more errors of both types. Because participants now have to monitor for both the NoGo cue (digit 3) and the prospective cue (color blue), a more successful strategy is to attend to NoGo trials and prospective cues more accurately on a trial-by-trial basis and respond independently to each item. This item-specific focus ultimately results in a decreased autoregressive parameter ϕ. This latter strategy seems to be implemented by participants in the second block of PGNG (and already in the second half of the first block of PGNG), as indicated by a higher amplitude of fluctuations and a lower autoregressive parameter value compared to the first PGNG block.

In PGNG, increased net IIV may thus result from two separate processes. First, as in CGNG, one part of net IIV in PGNG may reflect random process fluctuation. Second, the remaining part of increased net IIV might represent functional adaptability to the newly imposed requirements in PGNG. In addition, we see a change in performance pattern between the first and second block of PGNG. First, both inhibition and prospective errors decreased in the second block, indicating there was space for performance improvement in the first block. Second, while the amplitude of fluctuations remained stable across blocks, the autoregressive parameter was quite high in the first half of the first block of PGNG and decreased at the end of the block and in the second block, indicating less coherence in response pattern despite similar net IIV. Taken together, this confirms our expectation that at least some aspects of variability may be beneficial to performance in PGNG.

To summarize our interpretation of the two IIV components in these data, we suggest that a high autoregressive parameter ϕ in CGNG1 and in the first half of PGNG1 indicates exploration of best possible strategies during early phases of task acquisition, while net-IIV, as indicated by *iSD*, reflects detrimental random processes in the classical version of GNG. In turn, increased net IIV in both blocks of PGNG and decreased time-dependent autoregressive ϕ parameter in the second block both reflect a beneficial functional adaptability. In sum, these results confirm that mean level and net and time-dependent IIV in RTs provide different and complementary information in respect to task performance.

Our interpretations are coherent with the results from Loft and colleagues and Ihle and colleagues [36,64]. Because accuracy in the PM task used by Loft and colleagues was rather low and net IIV had a positive effect on PM performance, net IIV appears to reflect functional diversity and/or functional adaptability in their data. In contrast, PM performance in Ihle and colleagues was very close to optimal and net IIV had a detrimental effect on PM performance, reflecting random process fluctuations. In the end, these results illustrate the complexity and multiple aspects of IIV effects on cognitive processes and performance.

### 4.4. Limitations and Perspectives

First, it is not clear whether the divergent effects of random process fluctuations or functional adaptability in PGNG occur within the same individual, reflecting competing parallel processes, or across different individuals, reflecting interindividual differences in prioritizing one task over the other. Further studies are therefore needed to clarify under which circumstances net and time-dependent IIV appear to be adaptive strategies, and systematically examine how random process fluctuation and functional adaptability can simultaneously co-occur in the same task. If both effects add up in the same individual, methodological and statistical tools to disentangle their respective effects on *iSD* need to be further studied. From a methodological standpoint, manipulating instructions in order to clearly tell participants which task to prioritize over the other might help to clarify the effect of *iSD* on performance. From a statistical perspective, the conjoint combination of *iSD* and ϕ patterns appears to be a promising tool in that regard. We would predict that high *iSD* coupled with low ϕ would indicate nonadaptive RT strategies in simple tasks, but beneficial strategies in more complex or dual tasks. An additional lead would also be to classify participants into high versus low performers and to test for differential effects of *iSD* and ϕ in both groups.

Second, given that involvement of executive functions in PM seems to be exacerbated in older adults in comparison to younger adults [47], the present study could be extended to younger adults in order to clarify whether these results generalize to other age groups.

Third, the present study analyzed IIV in time series for RT only. Results could be extended to IIV in accuracy using an autoregressive model for binary data (e.g., [98]). These models would provide valuable complementary information in terms of regularity in response correctness, and potentially help to disentangle respective effects of net and time-structured IIV.

Fourth, we used raw IIV indices in this study. Ihle and colleagues analyzed costs to IIV and RT [64], and this methodological approach may provide leads to clarify which task participants might prioritize over the other. This could also help disentangle opposite effects of IIV on PM performance. We would predict that participants whose ϕ parameter decreased more between blocks 1 and 2 of PGNG would have better performance in the latter than the former.

Fifth, as previously proposed, the absence of a predictive effect of IIV on PM performance may result from the fact that automatic processes for cue detection might be sufficient for successful performance in PGNG. Indeed, despite requiring more cognitive control than CGNG, it appears that PGNG is not particularly taxing on executive resources, as shown by the particularly high PM accuracy rate. Because PGNG has the advantage of presenting a relatively simple design as compared to most PM tasks, this task was well suited for a first application of DSEM analysis. However, given that IIV appears to reflect controlled processes, PGNG might not be the most adequate task to reveal effects of IIV on PM performance. We therefore propose some methodological modifications to the task in order to increase controlled process task requirements. A first possibility is to use a less distinctive prospective cue, such as a particular color of frame in the periphery of the screen or a change in background pattern. Furthermore, because participants have to press an additional key for the prospective trials (space bar), in addition to the usual key-press for the ongoing task (J key) in PGNG, prepotent response inhibitory control is not directly required during the intention execution phase in PGNG. Critically, Bisiacchi and colleagues showed that prepotent inhibition response is particularly involved in PM performance when inhibitory control is directly required in intention execution (i.e., when participants have to inhibit the usual ongoing task response in order to press an alternative key for the prospective trials) [99] (see also [42]). We therefore expect the relationship between prepotent response inhibition and PM performance to be stronger in a paradigm where participants have to refrain from pressing the usual ongoing task response and instead press another response key for prospective trials only. As also suggested by Verbruggen and Logan, GNG itself might not be the best paradigm to measure controlled processes in prepotent response inhibition [100]. Because there is a consistent mapping between stimuli and response (Go or NoGo trials), with time, successful inhibition of the motor response in NoGo trials can rely on automatic processes too. The authors therefore recommend using a stop-signal paradigm, a task in which the stimulus-response mapping is inconsistent, instead of GNG, or reversing stimulus-response mapping between blocks (i.e., not always associating NoGo trials with the digit 3, but changing cue digits across blocks). However, we note that the stop-signal task, as the GNG task used here, might imply a strong motor component, which may diminish generalization to other PM tasks with inferior motor components. To conclude, using PGNG, we were able to underscore a predictive effect of inhibition on PM performance, but we expect this relation to be stronger and to strengthen the effect of IIV on PM performance with some task modifications aimed at increasing task requirements for controlled processes.

Sixth, the main indices we used to address our hypothesis are potentially quite different with respect to their reliability. As pointed out by past work (e.g., [85,101,102]), it is generally the case that in RT data the intraindividual mean is more reliable than the *iSD*. Indeed, the correlation between the indices estimated across the two blocks of the same tasks were r = 0.83, 0.68, and 0.41 for μ, *iSD*, and ϕ in CGNG, and 0.78, 0.65, and 0.45 in PGNG. This may point to a decreasing level of reliability going from μ to the *iSD* and ϕ, as was similarly obtained by Ghisletta and colleagues [16]. Yet, simulation work (e.g., [101,103]) has shown that with over 100 trials, one can reliably estimate *iSD* and ϕ. Given that here we worked with blocks of 117 trials, we suppose that our *iSD* estimates were reliable enough for inclusion in the extended DSEM models. As a matter of fact, *iSD* and ϕ were found to be significant predictors of commission errors in multiple blocks. In sum, while we recognize that the indices do vary with respect to measurement reliability, we do not think this feature alone explains our results.

Finally, we relied on DSEM to assess the respective contributions of mean level of RT as well as both net and time-structured aspects of IIV in within-task cognitive performance. This modeling framework lacks reliable goodness-of-fit indices, in both absolute and relative terms. In particular, at present the deviance information criterion (DIC) cannot be used to compare models directly, as is usually done in SEM [70]. We are sure that in the near future, model comparison will become possible also for DSEM.

## 5. Conclusions

Results of the present study indicate that higher RT latency and increased net variability are consistently associated with increased inhibition failure, while coherence in RT pattern predicts inhibition performance only when the task is novel. In turn, inhibition failure significantly predicts PM errors therein, corroborating the current PM literature on the involvement of prepotent response inhibition in successful PM processes. However, we did not find any effect of IIV on PM performance. In conclusion, the present work allowed us to highlight the multiple facets of IIV, in both its adaptive and detrimental aspects, in a classical inhibition task and in its prospective version. Our results further suggest that IIV should be considered not solely as reflecting inefficient sustained cognitive processes, but also as efficiently exploring strategies to attain and restore optimal performance [95]. In that regard, examining time-dependent aspects of IIV besides amplitude proved insightful. We encourage future developmental cognitive researchers to model time-dependent IIV as a window to deepening their understanding of dynamic cognitive processes and possible trade-off processes between competing tasks.

## Figures and Tables

**Table 1:** Means and standard deviations (*M, SD*) for commission errors and prospective omissions in blocks 1 and 2 of CGNG and PGNG.

Error Type	Block			
	CGNG1	CGNG2	PGNG1	PGNG2
Commission errors	1.18 (1.6)	1.03 (1.15)	3.61 (2.49)	2.48 (1.79)
Prospective omissions	na	na	1.53 (2.17)	0.75 (1.58)

Note: The maximum number of each type of error for each block is 13.

**Table 2:** Posterior means (and 95% credible intervals) of fixed effects, random effect variances, and correlations between random effects estimated from models M1 to M4. Mean fixed effects and random variances are shown in raw metrics; correlations and regression weights are shown in within-level standardized metrics.

Parameter	Block (Model)			
	CGNG1 (M1)	CGNG2 (M2)	PGNG1 (M3)	PGNG2 (M4)
$\gamma_{µ}$	6.25 * (6.22 to 6.28)	6.22 * (6.19 to 6.25)	6.38 * (6.35 to 6.41)	6.33 * (6.30 to 6.36)
$\upsilon_{µi}$	0.02 * (0.01 to 0.02)	0.02 * (0.01 to 0.02)	0.01 * (0.01 to 0.02)	0.02 * (0.01 to 0.02)
$\gamma_{\phi }$	0.30 * (0.26 to 0.33)	0.27 * (0.23 to 0.31)	0.23 * (0.19 to 0.21)	0.14 * (0.10 to 0.17)
$\upsilon_{\phi i}$	0.01 * (0.01 to 0.02)	0.02 * (0.01 to 0.03)	0.02 * (0.01 to 0.03)	0.01 * (0.01 to 0.02)
$\gamma_{iSD}$	0.15 * (0.14 to 0.16)	0.14 * (0.13 to 0.15)	0.20 * (0.19 to 0.21)	0.18 * (0.17 to 0.19)
$\upsilon_{iSDi}$	0.00 * (0.00 to 0.01)	0.00 * (0.00 to 0.01)	0.00 * (0.00 to 0.01)	0.00 * (0.00 to 0.01)
*r*$\left(\upsilon_{µi}-\upsilon_{\phi i}\right)$	0.22 (−0.10 to 0.47)	0.18 (−0.10 to 0.43)	−0.18 (−0.48 to 0.13)	0.09 (−0.25 to 0.39)
*r*$\left(\upsilon_{µi}-\upsilon_{iSDi}\right)$	0.11 (−0.12 to 0.31)	0.05 (−0.17 to 0.27)	0.11 (−0.12 to 0.33)	0.14 (−0.08 to 0.34)
*r*$\left(\upsilon_{\phi i}-\upsilon_{iSDi}\right)$	0.65 * (0.40 to 0.83)	0.29 * (0.03 to 0.52)	0.33 * (0.02 to 0.59)	−0.11 (−0.40 to 0.17)

Note: The parameters , $\gamma_{µ}$, $\gamma_{\phi }$, $\gamma_{iSD}$ are the fixed effects, while the parameters $\upsilon_{µi}$, $\upsilon_{\phi i}$, $\upsilon_{iSDi}$ are the random variances of the within-person mean RT, the order-1 autoregressive parameter, and the intraindividual standard deviation, respectively. Correlations between random effects at level 2 are denoted by *r*$\left(\upsilon_{µi}-\upsilon_{\phi i}\right)$, *r*$\left(\upsilon_{µi}-\upsilon_{iSDi}\right)$, and *r*$\left(\upsilon_{\phi i}-\upsilon_{iSDi}\right)$. * 95% CI does not include 0.

**Table 3:** Posterior means (and 95% CI) of intercepts and regression weights of $CE_{i}$, on $\mu_{i}$, $iSD_{i}$, and $\phi_{i}$, estimated for models 5 to 8. Estimates are shown in within-level standardized metrics.

Parameter	Block (Model)			
	CGNG1 (M5)	CGNG2 (M6)	PGNG1 (M7)	PGNG2 (M8)
$\beta_{0}$	11.15 * (1.74 to 19.51)	11.27 (−0.86 to 23.30)	14.94 * (2.78 to 26.53)	15.46 * (5.31 to 24.03)
$\beta_{µ}$	−0.27 *(−0.48 to −0.05)	−0.20 * (−0.40 to −0.00)	−0.27 * (−0.50 to –0.04)	−0.32 * (−0.51 to −0.10)
$\beta_{\phi }$	−0.39 * (−0.93 to −0.00)	0.08 (−0.18 to 0.33)	−0.24 (−0.56 to 0.08)	0.16 (−0.15 to 0.45)
$\beta_{iSD}$	0.79 * (0.50 to 1.33)	0.42 * (0.21 to 0.59)	0.31 * (0.07 to 0.55)	0.20 (−0.00 to 0.40)
$R_{CE}^{2}$	0.45	0.25	0.16	0.17

Note: The parameters $\beta_{0},\;\;\beta_{µ}$,$\beta_{\phi }$, $\beta_{iSD}$ are the respective intercepts and regression weights of commission errors on $\mu_{i}$, $iSD_{i}$, and $\phi_{i}$, the order-1 autoregressive parameter. $R_{CE}^{2}$ = between-level *R^2^* of commission errors. * 95% CI does not include 0. Full version of the table, including all estimates, is available in Table A1.

## Appendix A

**Table A1:** Posterior means (and 95% credible intervals) of fixed effects, random effect variances, correlations between random effects, intercepts and regression weights of $CE_{i}\;$ on $\mu_{i}$, $iSD_{i}$ and $\phi_{i}$ estimated for models 5 to 8. Mean fixed effects and random variances are shown in raw metrics while correlations and regression weights are shown in within-level standardized metrics.

Parameter	Block (Model)			
	CGNG1 (M5)	CGNG2 (M6)	PGNG1 (M7)	PGNG2 (M8)
$\gamma_{µ}$	6.25 * (6.22 to 6.28)	6.22 * (6.19 to 6.25)	6.38 * (6.35 to 6.41)	6.33 * (6.30 to 6.36)
$\upsilon_{µi}$	0.02 * (0.01 to 0.04)	0.02 * (0.01 to 0.02)	0.01 * (0.01 to 0.02)	0.02 * (0.01 to 0.03)
$\gamma_{\phi }$	0.29 * (0.25 to 0.33)	0.27 * (0.23 to 0.31)	0.23 * (0.20 to 0.27)	0.14 * (0.10 to 0.17)
$\upsilon_{\phi i}$	0.01 * (0.01 to 0.19)	0.02 * (0.01 to 0.03)	0.02 * (0.01 to 0.02)	0.01 * (0.01 to 0.02)
$\gamma_{iSD}$	0.15 * (0.14 to 0.16)	0.14 * (0.13 to 0.15)	0.20 * (0.19 to 0.21)	0.18 * (0.17 to 0.19)
$\upsilon_{iSDi}$	0.00 * (0.00 to 0.01)	0.00 * (0.00 to 0.01)	0.00 * (0.00 to 0.01)	0.00 * (0.00 to 0.01)
*r*$\left(\upsilon_{µi}-\upsilon_{\phi i}\right)$	0.23 (−0.07 to 0.72)	0.17 (−0.11 to 0.43)	−0.18 (−0.47 to 0.15)	0.09 (−0.24 to 0.39)
*r*$\left(\upsilon_{µi}-\upsilon_{iSDi}\right)$	0.13 (−0.11 to 0.64)	0.05 (−0.17 to 0.26)	0.11 (−0.11 to 0.32)	0.13 (−0.10 to 0.35)
*r*$\left(\upsilon_{\phi i}-\upsilon_{iSDi}\right)$	0.68 * (0.42 to 0.91)	0.30 * (0.02 to 0.53)	0.34 * (0.06 to 0.60)	−0.11 (−0.40 to 0.19)
$\beta_{0}$	11.15 * (1.74 to 19.51)	11.27 (−0.86 to 23.30)	14.94 * (2.78 to 26.53)	15.46 * (5.31 to 24.03)
$\beta_{µ}$	−0.27 * (–0.48 to −0.05)	−0.20 * (−0.40 to −0.00)	−0.27 * (−0.50 to −0.04)	−0.32 * (−0.51 to −0.10)
$\beta_{\phi }$	−0.39 * (−0.93 to −0.00)	0.08 (−0.18 to 0.33)	−0.24 (−0.56 to 0.08)	0.16 (−0.15 to 0.45)
$\beta_{iSD}$	0.79 * (0.50 to 1.33)	0.42 * (0.21 to 0.59)	0.31 * (0.07 to 0.55)	0.20 (−0.00 to 0.40)
$R_{CE}^{2}$	0.45	0.25	0.16	0.17

Note: The parameters $\gamma_{µ}$, $\gamma_{\phi }$, $\gamma_{iSD}$ are the fixed effects, while the parameters $\upsilon_{µi}$, $\upsilon_{\phi i}$, $\upsilon_{iSDi}$ are the random variances of the within-person mean RT, the order-1 autoregressive parameter, and the intraindividual standard deviation, respectively. Correlations between random effects at level 2 are denoted by *r*$\left(\upsilon_{µi}-\upsilon_{\phi i}\right)$, *r*$\left(\upsilon_{µi}-\upsilon_{iSDi}\right)$, and *r*$\left(\upsilon_{\phi i}-\upsilon_{iSDi}\right)$. $\beta_{0}$, $\beta_{µ}$, $\beta_{\phi }$, $\beta_{iSD}$ are the respective intercepts and regression weights of commission errors on $\mu_{i}$, ${iSD}_{i}$, and $\phi_{i}$. $R_{\mathrm{CE}}^{2}$ = between-level R*^2^* of commission errors. * 95% CI does not include 0.

## Appendix B

**Table A2:** Posterior means (and 95% credible intervals) of fixed effects, random effect variances, correlations between random effects, and regression weights of $CE_{i}\;$ on $\mu_{i}$ and $\phi_{i}$ estimated for models 5bis. Mean fixed effects and random variances are shown in raw metrics, while correlations and regression weights are shown in within-level standardized metrics.

Parameter	CGNG1 (M5bis)
$\gamma_{µ}$	6.25 * (6.23–6.28)
$\upsilon_{µi}$	0.02 * (0.01–0.03)
$\gamma_{\phi }$	0.31 * (0.28–0.34)
$\upsilon_{\phi i}$	0.01 * (0.01–0.02)
*r*$\left(\upsilon_{µi}-\upsilon_{\phi i}\right)$	0.27 (−0.06 to 0.56)
$\beta_{µ}$	−0.28 * (−0.49 to −0.05)
$\beta_{\phi }$	0.11 (−0.19 to 0.38)
$R_{CE}^{2}$	0.09

Note: The parameters $\gamma_{µ}$ and $\gamma_{\phi }$ are the fixed effects, while the parameters $\upsilon_{µi}$ and $\upsilon_{\phi i}$ are the random variances of the within-person mean RT and the order-1 autoregressive parameter, respectively. Correlation between random effects at level 2 is denoted by *r*$\left(\upsilon_{µi}-\upsilon_{\phi i}\right)$. $\beta_{µ}$ and $\beta_{\phi }$ are the respective regression weights of commission errors on $\mu_{i}$ and $\phi_{i}$. $R_{\mathrm{CE}}^{2}$ = between-level R*^2^* of commission errors. * 95% CI does not include 0.

**Table A3:** Posterior means (and 95% credible intervals) of fixed effects, random effect variances, correlations between random effects, intercepts, and regression weights of $CE_{i}\;$ on $\mu_{i}$, $iSD_{i}$, and $\phi_{i}$ estimated for the first and second half of PGNG1 trials. Mean fixed effects and random variances are shown in raw metrics, while correlations and regression weights are shown in within-level standardized metrics.

Parameter	Block	
	PGNG1—First Half of Block	PGNG1—Second Half of Block
$\gamma_{µ}$	6.40 * (6.37 to 6.43)	6.33 * (6.30 to 6.36)
$\upsilon_{µi}$	0.01 * (0.01 to 0.02)	0.02 * (0.01 to 0.02)
$\gamma_{\phi }$	0.24 * (0.20 to 0.29)	0.14 * (0.10 to 0.17)
$\upsilon_{\phi i}$	0.02 * (0.01 to 0.03)	0.01 * (0.00 to 0.02)
$\gamma_{iSD}$	0.21 * (0.20 to 0.22)	0.18 * (0.17 to 0.19)
$\upsilon_{iSDi}$	0.00 * (0.00 to 0.01)	0.00 * (0.00 to 0.01)
*r*$\left(\upsilon_{µi}-\upsilon_{\phi i}\right)$	−0.03 (−0.41 to 0.35)	−0.16 (−0.56 to 0.30)
*r*$\left(\upsilon_{µi}-\upsilon_{iSDi}\right)$	0.25 * (0.02 to 0.46)	0.01 (−0.22 to 0.23)
*r*$\left(\upsilon_{\phi i}-\upsilon_{iSDi}\right)$	0.48 * (0.13 to 0.77)	0.08 (−0.32 to 0.49)
$\beta_{0}$	12.46 (−2.38 to 30.40)	15.46 * (5.31 to 24.03)
$\beta_{µ}$	−0.22 (−0.57 to 0.06)	−0.20 (−0.43 to 0.09)
$\beta_{\phi }$	−0.48 * (−1.06 to −0.07)	0.31 (−0.05 to 0.76)
$\beta_{iSD}$	0.41 * (0.10 to 0.97)	0.33 * (0.07 to 0.54)
$R_{CE}^{2}$	0.23	0.30

Note: The parameters $\gamma_{µ}$, $\gamma_{\phi }$, $\gamma_{iSD}$ are the fixed effects, while the parameters $\upsilon_{µi}$, $\upsilon_{\phi i}$, $\upsilon_{iSDi}$ are the random variances of the within-person mean RT, the order-1 autoregressive parameter, and the intraindividual standard deviation, respectively. Correlations between random effects at level 2 are denoted by *r*$\left(\upsilon_{µi}-\upsilon_{\phi i}\right)$, *r*$\left(\upsilon_{µi}-\upsilon_{iSDi}\right),$ and *r*$\left(\upsilon_{\phi i}-\upsilon_{iSDi}\right)$. $\beta_{0}$, $\beta_{µ}$, $\beta_{\phi }$, $\beta_{iSD}$ are the respective intercepts and regression weights of commission errors on $\mu_{i}$, ${iSD}_{i}$, and $\phi_{i}$. $R_{\mathrm{CE}}^{2}$ = between-level *R**^2^* of commission errors. * 95% CI does not include 0.
